# Molecular Epidemiology and Phylogenetic Analysis of Human Adenovirus Caused an Outbreak in Taiwan during 2011

**DOI:** 10.1371/journal.pone.0127377

**Published:** 2015-05-18

**Authors:** Yung-Cheng Lin, Po-Liang Lu, Kuei-Hsiang Lin, Pei-Yu Chu, Chu-Feng Wang, Jih-Hui Lin, Hsin-Fu Liu

**Affiliations:** 1 Department of Bioscience and Biotechnology, National Taiwan Ocean University, Keelung, Taiwan; 2 Department of Medical Research, Mackay Memorial Hospital, Taipei, Taiwan; 3 Division of Infectious Diseases, Department of Internal Medicine, Kaohsiung Medical University Hospital, Kaohsiung, Taiwan; 4 Department of Clinical Laboratory, Kaohsiung Medical University, Kaohsiung, Taiwan; 5 Department of Medical Laboratory Science and Biotechnology, Kaohsiung Medical University, Kaohsiung, Taiwan; 6 Department of Laboratory Medicine, Kaohsiung Medical University Hospital, Kaohsiung Medical University, Kaohsiung, Taiwan; 7 Center for Research, Diagnostics and Vaccine Development, Centers for Disease Control, Taipei, Taiwan; 8 Center for General Education, National Taipei University of Nursing and Health Sciences, Taipei, Taiwan; National Institute of Health, ITALY

## Abstract

An outbreak of adenovirus has been surveyed in Taiwan in 2011. To better understand the evolution and epidemiology of adenovirus in Taiwan, full-length sequence of hexon and fiber coapsid protein was analyzed using series of phylogenetic and dynamic evolution tools. Six different serotypes were identified in this outbreak and the species B was predominant (HAdV-3, 71.50%; HAdV-7, 15.46%). The most frequent diagnosis was acute tonsillitis (54.59%) and bronchitis (47.83%). Phylogenetic analysis revealed that hexon protein gene sequences were highly conserved for HAdV-3 and HAdV-7 circulation in Taiwan. However, comparison of restriction fragment length polymorphism (RFLP) analysis and phylogenetic trees of fiber gene in HAdV-7 clearly indicated that the predominant genotype in Taiwan has shifted from 7b to 7d. Several positive selection sites were observed in hexon protein. The estimated nucleotide substitution rates of hexon protein of HAdV-3 and HAdV-7 were 0.234×10^-3^ substitutions/site/year (95% HPD: 0.387~0.095×10^-3^) and 1.107×10^-3^ (95% HPD: 0. 541~1.604) respectively; those of the fiber protein of HAdV-3 and HAdV-7 were 1.085×10^-3^ (95% HPD: 1.767~0.486) and 0.132×10^-3^ (95% HPD: 0.283~0.014) respectively. Phylodynamic analysis by Bayesian skyline plot (BSP) suggested that using individual gene to evaluate the effective population size might possibly cause miscalculation. In summary, the virus evolution is ongoing, and continuous surveillance of this virus evolution will contribute to the control of the epidemic.

## Introduction

Human adenoviruses (HAdVs) are double-stranded non-enveloped DNA viruses belonging to the family *Adenoviridae*, genus *Mastadenovirus*. More than sixty serotypes of HAdVs have been recognized and classified into seven species (A-G) based on genome sequencing, phylogenetic and biological characteristics [1, 2]. Capsid proteins such as hexon and fiber in HAdV play a critical role of entry into cell and immune response as well [3]. Antigenic and genetic variability of these regions can cause epidemics or outbreaks [4–6]. HAdV are implicated in a wide range of human diseases, including respiratory diseases, conjunctivitis, cystitis and gastroenteritis. Acute respiratory tract infection (ARTI) is a serious threat to infant and child who usually required hospitalization. Species B (HAdV-3, 7, 14, 55), C (HAdV-1, 2, 5, 6) and E (HAdV-4) are frequently isolated from pediatric patients with ARTI [4, 6–8]. In particular the HAdV-7 is frequently associated with the severe ARTI such as lethal pneumonia or bronchopneumonia [9, 10]

HAdV is one of the major pathogens of the ARTI in Taiwan [11]. A total of 3 outbreaks caused by this virus had been detected in Taiwan since 1999. HAdV-7 was responsible for the outbreak in 1999, HAdV-4 in 2000~2001, and HAdV-3 in 2004~5 [12, 13]. After the outbreak in 1999, isolation rate of HAdV-7 became lower. However, HAdV-3 was still the most common serotype during the past two decades [14]. In 2011, clinical isolates of adenovirus was significantly increased implying an outbreak of HAdV. To investigate the predominant serotype of the virus in this outbreak, and to reveal whether the predominant strains have antigenic or sequence variation in the hexon and/or fiber capsid protein gene, we conducted a comprehensive phylogenetic and evolutionary analysis. The clinical features of the adenovirus infections were also analyzed.

## Materials and Methods

### Ethics Statement and Study Design

A total of 207 isolates used in this study were stratified random sampling depending on number of monthly adenovirus positive case from positive stocks collected during the outbreak in 2011. Virus was isolated from either nasopharyngeal aspirate or throat swabs from children with ARTIs in Kaohsiung Medical University Chung-Ho Memorial Hospital, and then grown in H292 and A549 cells (purchased from American Type Culture Collection). The study of ethical approval was obtained from Kaohsiung Medical University Hospital Institutional Review Board (KMUH-IRB-980344). This was a retrospective study without intervention or obtaining extra clinical specimens. All samples were de-identified and analyzed anonymously, so informed consent was waived. The Institutional Review Board of Kaohsiung Medical University Hospital also approved the waiving of informed consent. Statistical analyses of the correlation between serotypes and clinical data were using JMP software (Version 8). The statistical significance was set at the level of p< 0.05.

### Serotype and genotype classification

Viral DNA was extracted using QIAamp DNA Mini Kit (Qiagen, Santa Clara, CA). For restriction fragment length polymorphism (RFLP) analysis, DNA extraction was according to the traditional phenol/chloroform/isoamyl alcohol (25:24:1) extraction methods [15]. DNA was stored at −80°C until use. The primer pair AdnU-S’ (5’-TTCCCCATGGCNCACAACAC-3’) and AdnU-A (5’-GCCTCGATGACGCCGCGGTG-3’) were used to amplify a 956-bp product from the hexon region[16]. PCR products were subjected to sequence assay and were used for the identification of serotypes. For genotyping, aliquots containing1–2 μg of viral DNA were digested with 10–15 U of BamHI, Bcl I, BstE II and Bgl II (Promega, Madison, WI, USA) according to the manufacturer’s instructions. Digested products were electrophoresis on 0.8% agarose gel containing SYBR Green I (Invitrogen, Ltd.) and run for 16 hr at 50 V in TBE buffer. The RFLP patterns were identified according to nomenclature system developed by Li et al. and other modification [17, 18].

### PCR amplification and sequencing of hexon and fiber genes

We designed six primers to amplify the full length of hexon and fiber genes. Primers Ad-3F-F (5’-ACCTCACCCTCTTCCCAACT-3’), Ad-3F-R (5’-GAAGGGGGAGGCAAAATAAC-3’), Ad-7F-F (5’-GAAATTTTCTCCCAGCAGCA-3’), Ad-7F-R (5’-GAAGGGGGAGGCAAAATAAC-3’) were used to amplify the full length HAdV-3 and HAdV-7 fiber gene, respectively. The primer pair of B1-H-F (5’-GCAGCAGAGGAGAAAGGAAG-3’) and B1-H-R (5’-GACGATGGCTTTGAGCTCTT-3’) was used to amplify the whole hexon gene in both HAdV-3 and HAdV-7. The primer sequence and annealing temperature was shown in supplementary table (S1 Table).

PCR amplification was done by Sensoquest Labcycler (SensoQuest GmbH) with *Pfu* DNA polymerase (Promega Corporation, WI). PCR products were purified with QIAquick spin (Qiagen, Valencia, CA) columns and subject to direct sequencing by BigDye 3.1 Terminator Cycle Sequencing reagents on ABI Prism 3730 DNA Analyzer (Applied Biosystems, Forest City, CA).

### Phylogenetic and phylodynamic analysis

Full length of hexon and fiber genes were alignment by Muscle implemented in the MEGA 6 software [19, 20]. Likelihood mapping analysis was performed to evaluate the phylogenetic signal with TREE-PUZZLE software version 5.2 [21] The transition/transversion ratio, base frequencies, and α parameter of gamma distribution were estimated by TREE-PUZZLE software version 5.2. Phylogenetic trees were reconstructed with the neighbor-joining (NJ) and maximum likelihood (ML) methods using the MEGA 6 and PhyML 3.0 [19, 22]. The robustness of the phylogenetic trees was statistically evaluated by bootstrap analysis with 1000 replicates. The bootstrap value >75% was considered to a monophyletic group.

The evolution rates and population size changes of HAdV were determined using Bayesian Markov Chain Monte Carlo (MCMC) method offered in BEAST v1.8.2 along with the BEAGLE library [23, 24]. The SRD06 nucleotide substitution model was used in all simulations as this model is recognized to provide better resolution for coding regions to Bayesian analysis [25]. The demographic model, included Bayesian skyline, constant size, exponential growth, logistic growth, and expansion growth was used to estimate evolutionary and population dynamic, under both molecular clock models (strict and relaxed) [23]. The best fit of demographic and clock model was estimated form model comparison by Akaike’s information criterion (AICM) in the Tracer program v1.6 [26]. The MCMC chains were run for sufficient time to achieve convergence (ESS>200). In addition, the uncertainty of parameter is estimated in 95% highest probability density (HPD). The Maximum Clade Credibility (MCC) tree was constructed by Tree Annotator v 1.7.4, with the 10% burn-in. The final of phylogenetic trees were edited by Figtree v1.4.2.

### Selection pressure of hexon and fiber protein genes

To determine the selection pressures on hexon and fiber protein of HAdV, we estimated the ratio of non-synonymous substitutions (dN) and synonymous substitutions (dS) per site based on ML trees under the appropriate substitution model, using the single likelihood ancestor counting (SLAC), fixed effects likelihood (FEL) methods with significance level on 0.05. Bayesian tests for selection acting on individual sites were using FUBAR with posterior probabilities on 0.95 [27]. The directional evolution in protein sequences (DEPS) test was using to detect selective sweeps [28]. Residues with a Bayes factor of >100 were reported as positively selected.

All methods were implemented in the HyPhy package and accessed through the Datamonkey web-server interface (http://www.datamonkey.org) [29, 30].

## Results

### Serotypes and clinical features

A total of 207 isolates were analysis by amplify partial hexon sequences and followed by BLAST search on NCBI database (http://blast.ncbi.nlm.nih.gov/Blast.cgi). All isolates were included in the clinical feature analysis. Among the 2011 outbreaks, six different serotypes were identified and the species B were the predominant strains (HAdV-3, 71.50%; HAdV-7, 15.46%) (Table 1). We only focus on these two major serotypes in this study.

**Table 1:** Demographic and clinical data for adenovirus-positive patients according to each serotype.

		Number of adenovirus serotypes (%)						
Variables	Total HAdV- positives		HAdV—1	HAdV—2	HAdV—3	HAdV—5	HAdV—6	HAdV—7
Serotype	207		6(2.89)	16(7.73)	148(71.50)	2(0.97)	3(1.45)	32(15.46)
Sex								
Male	82(60.0)		3(50.0)	5(31.25)	93(62.84)	1(50.0)	2(66.67)	23(65.63)
Female	125(40.0)		3(50.0)	11(68.75)	55(37.16)	1(50.0)	1(33.33)	11(34.38)
Age								
	5.12±4.08		2.33±1.37	2.53±1.31	5.21±3.76	3.75±3.18	3.0±0.0	6.77±5.79
Clinical presentation								
Length of hospitalization	6.21±3.15		5.33±3.39	5.56±1.93	6.40±3.30	5.0±0.0	6.0±1.00	5.91±3.14
Fever (°C)	38.88±2.87		38.57±0.96	38.96±0.93	39.13±0.94	39.05±0.49	39.17±0.29	37.72±0.6.92
Fever (days)	3.62±2.35		5.50±5.01	4.19±2.37	3.38±1.95	2.50±0.71	1.67±0.58	4.35±3.11
Fever	199(96.14)		6(100.00)	16(100.00)	142(95.95)	2(100.00)	3(100.00)	30(93.75)
Cough	158(76.33)		5(83.33)	13(81.25)	112(75.68)	2(66.67)	24(75.00)	158(76.33)
Rhinorrhoea	126(60.87)		4(66.67)	9(56.25)	91(61.49)	2(100.00)	2(66.67)	18(56.25)
Diarrhea	71(34.30)		0(0.00)	4(25.00)	52(35.14)	1(50.00)	1(33.33)	13(40.63)
Clinical presentation								
Acute tonsilitis	113(54.59)		4(66.67)	11(68.75)	81(54.73)	1(50.00)	3(100.00)	13(40.63)
Acute pharyngitis	22(10.63)		1(16.67)	1(6.25)	15(10.14)	0	0	5(15.63)
Acute sinusitis	36(17.39)		1(16.67)	3(18.75)	29(19.59)	0	0	3(9.38)
Bronchopneumonia	53(25.60)		2(33.33)	4(25.00)	37(25.00)	0	2(66.67)	8(25.00)
Bronchitis	99(47.83)		4(66.67)	5(31.25)	73(49.32)	2(66.67)	2(66.67)	13(40.63)
Pneumonia	26(12.56)		0	2(12.50)	21(14.19)	0	0	3(9.38)
Gastroenteritis	28(13.53)		0	2(12.50)	23(15.54)	0	0	3(9.38)

The most common symptoms were fever (96.14%), cough (76.33%) and rhinitis (60.87%). The most frequent diagnosis was acute tonsillitis (54.59%) and bronchitis (47.83%). The male-to-female ratio is 1.5:1; the mean age of the patients was 5.12 ± 4.08 years. The mean peak body temperature and length of hospitalization was 38.88 ± 2.87°C and 6.21±3.15 days respectively. The age distribution and fever days showed significant difference in patterns depending on the HAdV serotypes (*P* = 0.007and 0.032). Furthermore, HAdV-3 presented higher fever than that of HAdV-7(*P* = 0.011) (Table 1).

### Phylogenetic analysis of the hexon and fiber genes

**Fig 1:**
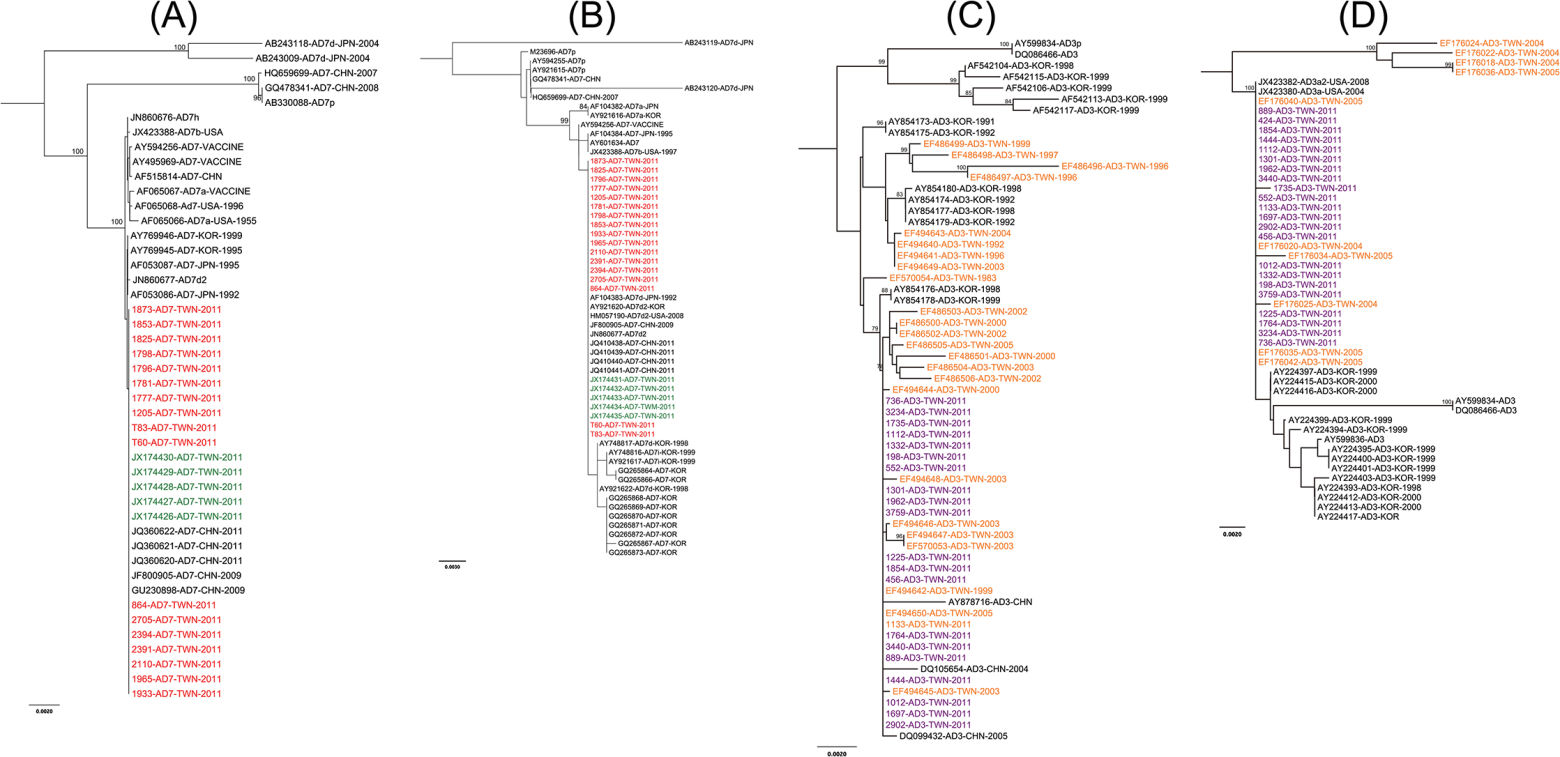
Phylogenetic analysis of the human adenovirus type 3 and type 7 hexon and fiber genes. The phylogenetic tree was inferred from HAdV-7 hexon gene (A), HAdV-7 fiber gene (B), HAdV-3 hexon gene (C) and HAdV-3 fiber gene (D). Tree topology was constructed using the neighbor-joining method. The topologic accuracy of the tree was evaluated by using 1,000 bootstrap replicates. Only bootstrap values greater than 75% are shown. Red and purple texts represent HAdV- 7 and 3 isolated in Taiwan in this outbreak, respectively. Orange text represents HAdV- 3 reference sequences from Taiwan. Green text was HAdV- 7 reference sequences from Northern Taiwan.

Likelihood mapping analysis showed that most of the quartets were located in the three corners of the triangle suggesting a treelike signal. Consistent tree topologies were observed by NJ and ML methods. The analyzed HAdV-7 hexon gene sequences were highly conserved and phylogenetic analysis showed they were clustered together with reference sequences from NCBI database and the strains isolated in same period from north Taiwan [31], except prototype and two 7d isolates from Japan in 2004 (with 96.3% and 95.4% identity respectively) (Fig 1A). For the fiber gene, our results showed that HAdV-7 in this outbreak were all classified into a single cluster with HAdV-7d2 and 7d rather than the previous predominant genotype 7b (Fig 1B). To confirm this observation, whole genome viral DNA was analyzed by RFLP and compared with the previous report [13, 17, 18]. These restriction patterns of Bam HI showed the HAdV-7 in this outbreak was belong to 7d or 7d2 genotype. Furthermore, three restriction enzymes (BstE II, Bcl I and Bgl II) conformed that they were not 7d2 genotype (S1 Fig). Based on these results we suggest the predominant of HAdV-7 in Taiwan has shifted from 7b to 7d genotype.

Similar results were observed from HAdV-3, all hexon and fiber gene sequences were highly conserved and also clustered together with our previously reported strains and other reference strains from Taiwan (Fig 1C and 1D). The viral nucleotide sequences determined in this study have been assigned with GenBank accession numbers KC456083 to KC456103 (HAdV-3 hexon genes), KC456104 to KC456125 (HAdV-3 fiber genes), KC456126 to KC456142 KC456125 (HAdV-7 fiber genes) and KC456143 to KC456159 (HAdV-7 hexon genes). The accession numbers of sequences used for phylogenetic and evolutionary analysis in this study were listed in supplementary table (S2 Table).

### Selection pressures in the HAdV- 3 and 7 surface proteins

Selection pressures of hexon and fiber protein in HAdV-3 and 7 were estimated by the dN/dS ratio. The criteria were 1) the ratio of dN/dS<1 as negative selection, 2) dN/dS = 1 as neutrality, and 3) dN/dS >1 as positive selection. The mean ratio of dN/dS in HAdV-3 and 7 hexon, fiber protein was 0.520, 0.534 and 0.092, 0.142, respectively (Table 2).

**Table 2:** Selection sites detected in hexon and fiber of HAdV- 3 and 7.

	Positively selected sites				No. of negatively selected sites			Mean d_N_/d_S_
	SLAC^a^	FEL^a^	FUBA^b^	DEPS^c^	SLAC	FEL	FUBA	
HAdV- 3								
Hexon	Non	649	137,205,649	22,205,254,299,326,386,417,429,439,649,651,652,667,675,714	2	5	4	0.520
Fiber	Non	Non	Non	Non	Non	3	3	0.534
HAdV- 7								
Hexon	146	Non	Non	443	6	44	27	0.092
Fiber	Non	Non	Non	104	Non	3	3	0.142

^a^ P value of <0.05.

^b^ Posterior probability of ≥0.95.

^c ^Bayes factor of >100

All of open reading frames contained negatively selected codons. The Hexon of HAdV-7 revealed relatively higher negatively selected codons than others. Positive selection was detected on HAdV-3 hexon protein at codon 649 by FEL method. Three codons (137, 205, 649) were detected by FUBA whereas other 15 codons were detected by DEPS.

For HAdV-7, two positive selection sites were detected in hexon protein of which codons 146 by SLAC method and codons 443 by DEPS. A positive selection site was detected in fiber at codons 104 by DEPS method.

### Phylodynamic of adenovirus

**Fig 2:**
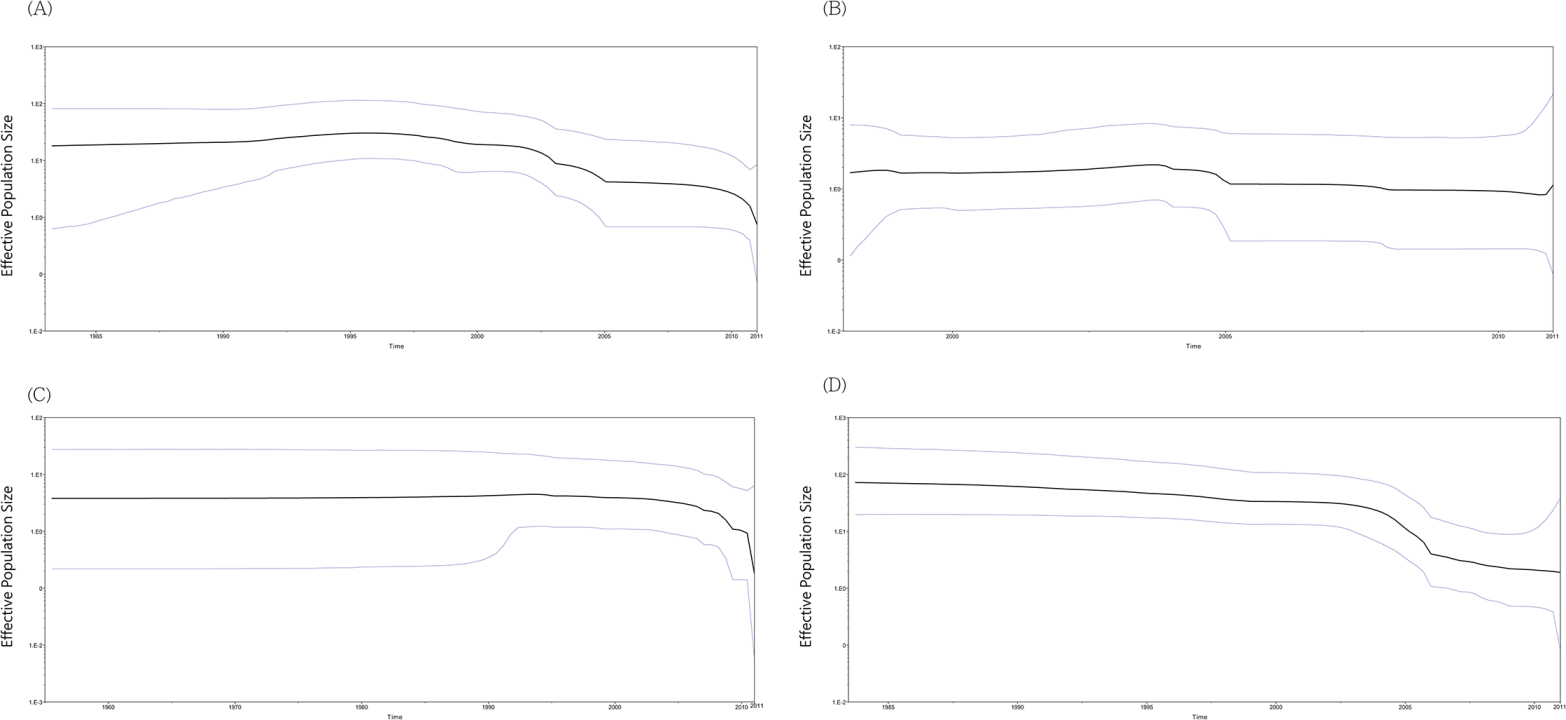
Bayesian skyline plot for complete hexon and fiber gene of the HAdV-3 and HAdV-7. The BSP were inferred from HAdV-3 hexon gene (A), HAdV-3 fiber gene (B), HAdV-7 hexon gene (C) and HAdV-7 fiber gene (D). The bold line represents the median estimate of the effective population size of infections through time, with the 95% HPD values shown within the blue line.

Phylodynamics of HAdV-3 and HAdV-7 was estimated by Bayesian skyline plot basis on hexon and fiber gene, respective. The genetic diversity of the HAdV-7 population remained steady until 2005, subsequently, the steep declined the effective population size lasted until 2011(Fig 2C and 2D). The result of hexon gene in HAdV-3 showed the population was declining after 1999 (Fig 2A). BSP constructed form HAdV-3 hexon, HAdV-7 hexon and fiber were represent downtrend for population dynamics (Fig 2A, 2C, and 2D). However, inconsistent results were found in the HAdV-3 fiber gene. That was steady after 2005 and showing upward trend at after 2010 (Fig 2B).

### Evolutionary rates and the most recent common ancestor (tMRCA) of HAdV- hexon and fiber genes

The uncorrelated lognormal relaxed clock model and Bayesian skyline was determined to be a better fit model for both hexon genes. For HAdV-7 fiber gene, the uncorrelated exponential relaxed and Bayesian skyline was the better fit model. For HAdV-3 fiber gene, the better fit model was uncorrelated lognormal relaxed clock model and exponential growth. All of the nucleotide substitution rates, evolutionary rates and tMRCA are summarized in Table 3. The estimated nucleotide substitution rate of hexon protein on HAdV-3 and HAdV-7 was 0.234×10^–3^ substitutions/site/year (95% HPD was 0.387~0.095×10^–3^) and 1.107×10^–3^ (95% HPD was 0.541~1.604), respectively. The nucleotide substitution rate of fiber protein on HAdV-3 and HAdV-7 was 1.085 ×10^–3^ (95% HPD: 1.767~0.486) and 0.132 ×10^–3^ (95% HPD: 0.283~0.014), respectively. The TMRCA of HAdV- 3 was dated to 1964 (95% HPD: 1918~1983) for hexon; the fiber was dated to 1995 (95% HPD: 1991~1998). The TMRCA of HAdV-7 hexon was dated to1949 (95% HPD: 1931~1955). However, the TMRCA of fiber gene was more than two hundred years (1788, 95% HPD: 1422~1984).

**Table 3:** Mean relative evolutionary rates for codon positions and times of most recent common ancestor (TMRCA) in hexon and fiber gene of Human adenovirus.

	TMRCA	Substitution rates (× 10^–3^)	Mean relative	SE of
	(calendar year)	subs/site/year	substitution rate	mean
HAdV- 3				
Fiber	1995	1.085		
	(1998~1991)	(1.767~0.486)		
1st codon position			1.211	1.8918E-3
(95% HPD)			(0.7317~1.673)	
2nd codon position			0.578	1.5242E-3
(95% HPD)			(0.2419~0.9669)	
3rd codon position			1.211	1.8817E-3
(95% HPD)			(0.7251~1.669)	
Hexon	1964	0.234		
	(1983~1918)	(0.387~0.095)		
1st codon position			0.711	9.1974E-4
(95% HPD)			(0.4913~0.9411)	
2nd codon position			0.818	1.1818E-3
(95% HPD)			(0.5521~1.1008)	
3rd codon position			1.471	1.1912E-3
(95% HPD)			(1.1008~1.7713)	
HAdV- 7				
Fiber	1788	0.132		
	(1422~1984)	(0.283~0.014)		
1st codon position			0.422	9.677 E-4
(95% HPD)			(0.1741~0.67)	
2nd codon position			0.504	1.0291 E-3
(95% HPD)			(0.2447~0.774)	
3rd codon position			2.074	1.2913 E-3
(95% HPD)			(1.7414~2.4109)	
Hexon	1949	1.107		
	(1931~1955)	(0.541~1.604)		
1st codon position			0.326	7.0944 E-4
(95% HPD)			(0.2036~0.4544)	
2nd codon position			0.25	6.8106 E-4
(95% HPD)			(0.1362~0.373)	
3rd codon position			2.424	9.0516 E-4
(95% HPD)			(2.2558~2.5802)	

The evolutionary rate of synonymous positions (3rd codon position) was significantly higher than that of nonsynonymous positions (1st and 2nd codon positions) in hexon and fiber genes, except the fiber gene of HAdV-3.

## Discussion

Previous studies suggested that viral genetic diversity cause by recombination was considered as main source of emerging outbreaks [30, 32–34]. For example, HAdV-7h, first isolated from the Buenos Aires, Argentina in 1987, had been reported to be highly virulent and predominant only in South America and Japan [8]. Sequence and phylogenetic analysis of HAdV-7h clearly indicated that it was an emerging virus and resulting from the recombination of HAdV-3 fiber [8].

During the last decade, emerging and/or re-emerging adenovirus cause several outbreaks worldwide, including Taiwan, Malaysia, China, the United States, Japan, France, Korea and Portugal [1, 4–6, 12, 13, 32, 35–37]. In 2011, a community outbreak of respiratory tract infections was observed in Taiwan and the majority of the patients required hospitalization. PCR and sequence of partial hexon gene showed the HAdV-3 and HAdV-7 were predominant and constituted this outbreak. The HAdV-3 and HAdV-7 also caused an outbreak in nearby countries China and Korea [38, 39]. The mean age of the patients of this outbreak was 5.12 ± 4.08 years. The male-to-female ratio (1.5:1) showed that HAdV infections affected more boys than girls. These clinical phenomenons are in consistent with previous studies [14, 40, 41]. However, the age distribution showed a significant different pattern depending on the HAdV serotypes, meaning that subgenus B was significantly older than that of subgenus C (*P* = 0.007). This observation was similar to that of HAdV-3 outbreak in Taiwan in 2004~2005 [12].

In Taiwan, HAdV-3a genotype was predominated during the 1983–1999, while HAdV-3a2 was predominated in 2001–2005 [14]. Comparison with phylogenetic trees and sequence alignments of fiber and hexon gene sequences from our previous data indicated that HAdV-3 was highly conserved with other Taiwan strains, and the predominant genotype did not change. According to our surveillance, HAdV-7 was not the major serotype circulating in Taiwan, but the HAdV-7b genotype was the predominant genotype of HAdV-7 in Taiwan [13]. Interestingly, our phylogenetic analysis showed that all the isolates from this outbreak were clustered with HAdV-7d2 from USA, HAdV-7d from Japan, and HAdV-7d and HAdV-7i form Korea (GenBank accession number HM057190, JN860677, AF104383, AF053087, AY921622 and AY7748816 respectively), rather than previous predominant HAdV-7b. This result suggested that the predominant genotype may have shifted. Further whole genome RFLP analysis supported this hypothesis.

The HAdV-7d, or a closely related variant, HAdV-7d2, was predominant genotype in Japan, Korea and China after 1984 [14, 42, 43], whereas HAdV-7b was predominant genotype in the same period in Taiwan till 2004. After the outbreak in 1999, HAdV-7 isolation rate was decreased in our and other contracted virology laboratories [31]. Interestingly, the transmission route of HAdV-7d that contributed to its emergence remained an important concern. One possible explanation was the frequent travels between Taiwan, China, Japan, Korea, and North American. We hypothesized that HAdV-7d genotype was introduced from an outside source resulting its emergence in 2011. This can be clearly seen in the MCC tree that all isolates form 2011 were clustered with isolates from China in the same period.

Phylodynamics of the hexon gene of HAdV-7 and HAdV-3 showed a downward trend of the effective population size in recent years. This suggested that genetic evolution of hexon gene for both HAdV-3 and 7 were stabilized. However, different results from hexon gene were found in HAdV-3 fiber gene. The effective population size showed on BSP was rise in 2011, which consistent with adenovirus outbreak in 2011.

The amino acid variation associated with positive selection was often observed in hexon gene. Hexon is the surface glycoprotein and thus is an accessible target to antibodies, which could explain the positive selection occurred in there.

For HAdV-3, the amino acid variation (G205V) located within HVR_3_ (hypervariable region) was detected as a positive selection site by FUBA and DEPS, while this nonsynonymous substitution was found in a new genotype of HAdV-3 either [44, 45]. The G205V variation might be beneficial to P649 H or R located within HVR_7_ also was positive selection site detected by FEL, FUBA, and DEPS. This variation was found in HAdV-3a and HAdV-3a2 subgroups in Taiwan in 1996~1999 and 2002~2005. For HAdV-7, positive selection site was observed in HVR_1_ of hexon protein at T146 (436_437 del AC) by SLAC. This amino acid residue only existed in the prototype strain but was deleted in all other HAdV-7 genotypes [46]. This deletion might conducive to HAdV-7 survival. On the other hand, another positive selection at L443Q was found by DEPS. The substitution was from Leu in HAdV-7b to Gln in Ad7d and affected the hydropathic characteristic (hydrophobic to hydrophilic) [46]. HAdV-7d was replaced HAdV-7b as the predominant circulating virus in our neighboring countries. Therefore, we suggest the L443Q was important codon for HAdV-7d expansion. The relatively low *d*N/*d*S ratios in HAdV-7 hexon gene implicated no strong selection occurred in HAdV-7.

This is the first report of dynamic evolution and selection pressure for Adenovirus in Taiwan. In this study, we found that the predominant genotype of HAdV-7 has changed. Although the evolution of the two major capsid proteins was steady, the virus evolution is ongoing under selection pressure. The acquisition of additional mutations in the future could lead to an antigenic drift and cause further outbreaks. In order to quickly respond to an outbreak caused by emergent or re-emergent adenovirus in the future, continuous surveillance of this virus evolution is necessary.

## Supporting Information

S1 Fig Restriction profiles of human adenovirus genome types after digestion with selected enzymes (Bam HI, Bcl I, BstE II and Bgl II) and DNA markers (λ Hind III and ϕX174 Hinc). M: molecular weight marker; Lane 1–1~1–4 was genotype 7b(2123/99) digestion with Bgl II, BstE II, Bcl I and Bam HI, respectively; Lane 2–1~2–4 was genotype 7d(1205/2011) digestion with Bgl II, BstE II, Bam HI and Bcl I, respectively; Lane 3–1~3–4 was genotype 7d(1777/2011) digestion with BstE II, Bgl II, Bam HI and Bcl I, respectively.
(EPS)

S1 Table Primers used for amplification and sequencing hexon and fiber genes. (DOCX)

S2 Table The list of references strains used to phylogenetic and evolutionary analysis in this study. (DOCX)
