# Supplementary material for: Molecular Epidemiology and Phylogenetic Analysis of Human Adenovirus Caused an Outbreak in Taiwan during 2011
Source: PLoS One. 2015 May 18;10(5):e0127377. doi: 10.1371/journal.pone.0127377 (PMC4436380; doi:10.1371/journal.pone.0127377)
Supplement: S1 Table — (DOCX) [file pone.0127377.s002.docx]

S1 Table: Primers used for amplification and sequencing Hexon and Fiber genes.

| *Primer* | *Primer sequence (5’→ 3’)* | *Gene* | *Annealing temperature* | *Use* |
| --- | --- | --- | --- | --- |
| Ad-3F-F | ACCTCACCCTCTTCCCAACT | Fiber | 58 | PCR and sequencing for Ad 3 fiber gene |
| Ad-3F-R | GAAGGGGGAGGCAAAATAAC | Fiber |  |  |
| Ad-3F-2F | AAAAATGGAGGAATTGTTAATGGA | Fiber |  | Sequencing for Ad 3 fiber gene |
| Ad-3F-2R | ATGCAGTTGGCTTCTGGTTT | Fiber |  | Sequencing for Ad 3 fiber gene |
| Ad-7F-F | GAAATTTTCTCCCAGCAGCA | Fiber | 51 | PCR and sequencing for Ad 7 fiber gene |
| Ad-7F-R | GAAGGGGGAGGCAAAATAAC | Fiber |  |  |
| Ad-7F-2F | TGGCC TCCAG TGAAT CTAAT G | Fiber |  | Sequencing for Ad 7 fiber gene |
| Ad-7F-2R | TTTGACAGTTGGCTCTGGTG | Fiber |  | Sequencing for Ad 7 fiber gene |
| B1-H-F | GCAGCAGAGGAGAAAGGAAG | Hexon | 54 | PCR and sequencing for Ad 3 and Ad 7 hexon gene |
| B1-H-R | GACGATGGCTTTGAGCTCTT | Hexon |  |  |
| ADV(37)Hf2 | ATTCCGGCACAGCTTACAAT | Hexon |  | Sequencing for Ad 3 and Ad 7 hexon gene |
| ADV(37)Hf3 | GAAACTCCAGACAGYCATGTRG | Hexon |  | Sequencing for Ad 3 and Ad 7 hexon gene |
| ADV(37)Hf4 | GGMAACAAYCWGGCTATGGA | Hexon |  | Sequencing for Ad 3 and Ad 7 hexon gene |
| ADV(37)Hf5 | CATGGCTCACAACACMGCTT | Hexon |  | Sequencing for Ad 3 and Ad 7 hexon gene |
| ADV(37)Hf6 | ACAAGGATCGCATGTACTCYTTT | Hexon |  | Sequencing for Ad 3 and Ad 7 hexon gene |
| ADV(37)Hr2 | GCTACGRTCGGTGGTCACAT | Hexon |  | Sequencing for Ad 3 and Ad 7 hexon gene |
| ADV(37)Hr3 | GGTCTTGCAAAAGACCCGTA | Hexon |  | Sequencing for Ad 3 and Ad 7 hexon gene |
| ADV(37)Hr4 | CTCGATGCCATGATTTTCAA | Hexon |  | Sequencing for Ad 3 and Ad 7 hexon gene |
| ADV(37)Hr5 | GCAAAGAATTTYTGAGGCACTT | Hexon |  | Sequencing for Ad 3 and Ad 7 hexon gene |
| ADV(37)Hr6 | ACTCATTTGGRSWCAACAGC | Hexon |  | Sequencing for Ad 3 and Ad 7 hexon gene |
| ADV(37)Hr7 | GGGATCCACCTCAAAAGTCA | Hexon |  | Sequencing for Ad 3 and Ad 7 hexon gene |
