# Supplementary material for: Molecular Epidemiology and Phylogenetic Analysis of Human Adenovirus Caused an Outbreak in Taiwan during 2011
Source: PLoS One. 2015 May 18;10(5):e0127377. doi: 10.1371/journal.pone.0127377 (PMC4436380; doi:10.1371/journal.pone.0127377)
Supplement: S2 Table — (DOCX) [file pone.0127377.s003.docx]

S2 Table： The list of references strains used to phylogenetic and evolutionary analysis in this study**.**

| Accession numbers | Country | Gene | Year of the specimen collected | Serotype |
| --- | --- | --- | --- | --- |
| AY599834 |  | Fiber |  | 3 |
| DQ086466 |  | Fiber |  | 3 |
| EF176034 | Taiwan | Fiber | 2005 | 3 |
| AY224397 | Korea | Fiber | 1999 | 3 |
| AY224415 | Korea | Fiber | 2000 | 3 |
| AY224416 | Korea | Fiber | 2000 | 3 |
| AY224399 | Korea | Fiber | 1999 | 3 |
| AY224394 | Korea | Fiber | 1999 | 3 |
| AY224395 | Korea | Fiber | 1999 | 3 |
| AY224400 | Korea | Fiber | 1999 | 3 |
| AY224401 | Korea | Fiber | 1999 | 3 |
| AY599836 | USA | Fiber | 1997 | 3 |
| AY224403 | Korea | Fiber | 1999 | 3 |
| AY224393 | Korea | Fiber | 1998 | 3 |
| AY224412 | Korea | Fiber | 2000 | 3 |
| AY224413 | Korea | Fiber | 2000 | 3 |
| AY224417 | Korea | Fiber |  | 3 |
| KC456106  (1112 – TWN) | Taiwan | Fiber | 2011 | 3 |
| KC456105  (1012 – TWN) | Taiwan | Fiber | 2011 | 3 |
| KC456108  (1133 – TWN) | Taiwan | Fiber | 2011 | 3 |
| KC456104  (1225 – TWN) | Taiwan | Fiber | 2011 | 3 |
| KC456107  (1301 – TWN) | Taiwan | Fiber | 2011 | 3 |
| KC456109  (1332 – TWN) | Taiwan | Fiber | 2011 | 3 |
| KC456110  (1444 – TWN) | Taiwan | Fiber | 2011 | 3 |
| KC456111  (1697 – TWN) | Taiwan | Fiber | 2011 | 3 |
| KC456113  (1764 – TWN) | Taiwan | Fiber | 2011 | 3 |
| KC456115  (1962 – TWN) | Taiwan | Fiber | 2011 | 3 |
| KC456116  (198 – TWN) | Taiwan | Fiber | 2011 | 3 |
| KC456114  (1854 – TWN) | Taiwan | Fiber | 2011 | 3 |
| KC456117  (2902 – TWN) | Taiwan | Fiber | 2011 | 3 |
| KC456118  (3234 – TWN) | Taiwan | Fiber | 2011 | 3 |
| KC456119  (3440 – TWN) | Taiwan | Fiber | 2011 | 3 |
| KC456120  (3759 – TWN) | Taiwan | Fiber | 2011 | 3 |
| KC456121  (424 – TWN) | Taiwan | Fiber | 2011 | 3 |
| KC456122  (456 – TWN) | Taiwan | Fiber | 2011 | 3 |
| KC456124  (736 – TWN) | Taiwan | Fiber | 2011 | 3 |
| KC456123  (552 – TWN) | Taiwan | Fiber | 2011 | 3 |
| KC456125  (889 – TWN) | Taiwan | Fiber | 2011 | 3 |
| KC456112  (1735 – TWN) | Taiwan | Fiber | 2011 | 3 |
| EF176020 | Taiwan | Fiber | 2004 | 3 |
| EF176035 | Taiwan | Fiber | 2005 | 3 |
| EF176040 | Taiwan | Fiber | 2005 | 3 |
| EF176042 | Taiwan | Fiber | 2005 | 3 |
| JX423380 | USA | Fiber | 2004 | 3 |
| JX423382 | USA | Fiber | 2008 | 3 |
| EF176025 | Taiwan | Fiber | 2004 | 3 |
| EF176024 | Taiwan | Fiber | 2004 | 3 |
| EF176022 | Taiwan | Fiber | 2004 | 3 |
| EF176018 | Taiwan | Fiber | 2004 | 3 |
| EF176036 | Taiwan | Fiber | 2005 | 3 |
| JN860676 | Argentina | Fiber | 1987 | 7h |
| Z48954 | Argentina | Fiber | 1987 | 7h |
| AB243119 | Japan | Fiber | 2003 | 7 |
| AB243120 | Japan | Fiber | 2003 | 7 |
| AF104382 | Japan | Fiber |  | 7 |
| AY921616 | USA | Fiber |  | 7 |
| AY594256 |  | Fiber |  | 7 |
| AF104384 | Japan | Fiber | 1995 | 7 |
| AY601634 | USA | Fiber | 1997 | 7 |
| JX423388 | USA | Fiber | 1997 | 7 |
| AY748817 | Korea | Fiber | 1998 | 7 |
| AY921622 | Korea | Fiber | 1998 | 7 |
| AY748816 | Korea | Fiber | 1999 | 7 |
| AY921617 | Korea | Fiber | 1999 | 7 |
| 60265864 | Korea | Fiber | 2005 | 7 |
| GQ265866 | Korea | Fiber | 2005 | 7 |
| 60265868 | Korea | Fiber | 2006 | 7 |
| GQZ65869 | Korea | Fiber | 2006 | 7 |
| GQ265870 | Korea | Fiber | 2006 | 7 |
| GQ265871 | Korea | Fiber | 2006 | 7 |
| GQ265872 | Korea | Fiber | 2006 | 7 |
| GQ265867 | Korea | Fiber | 2006 | 7 |
| GQ265873 | Korea | Fiber | 2006 | 7 |
| KC456140  (864 – TWN) | Taiwan | Fiber | 2011 | 7 |
| KC456139  (2705 – TWN) | Taiwan | Fiber | 2011 | 7 |
| KC456138  (2394 – TWN) | Taiwan | Fiber | 2011 | 7 |
| KC456137  (2391 –TWN) | Taiwan | Fiber | 2011 | 7 |
| KC456136  (2110 – TWN) | Taiwan | Fiber | 2011 | 7 |
| KC456135  (1965 – TWN) | Taiwan | Fiber | 2011 | 7 |
| KC456134  (1933 – TWN) | Taiwan | Fiber | 2011 | 7 |
| KC456133  (1873 – TWN) | Taiwan | Fiber | 2011 | 7 |
| KC456132  (1853 – TWN) | Taiwan | Fiber | 2011 | 7 |
| KC456131  (1825 – TWN) | Taiwan | Fiber | 2011 | 7 |
| KC456130  (1798 – TWN) | Taiwan | Fiber | 2011 | 7 |
| KC456129  (1796 – TWN) | Taiwan | Fiber | 2011 | 7 |
| KC456128  (1781 – TWN) | Taiwan | Fiber | 2011 | 7 |
| KC456126  (1205 – TWN) | Taiwan | Fiber | 2011 | 7 |
| KC456127  (1777 – TWN) | Taiwan | Fiber | 2011 | 7 |
| KC456141  (T60 – TWN) | Taiwan | Fiber | 2011 | 7 |
| KC456142  (T83 – TWN) | Taiwan | Fiber | 2011 | 7 |
| AF104383 | Japan | Fiber | 1992 | 7 |
| AY921620 | Japan | Fiber |  | 7 |
| HM057190 | USA | Fiber | 2008 | 7 |
| JF800905- | China | Fiber | 2009 | 7 |
| JN860677 | USA | Fiber | 2009 | 7 |
| JQ410438 | China | Fiber | 2011 | 7 |
| JQ410439 | China | Fiber | 2011 | 7 |
| JQ410440 | China | Fiber | 2011 | 7 |
| JQ410441 | China | Fiber | 2011 | 7 |
| JX174431 | Taiwan | Fiber | 2011 | 7 |
| JX174432 | Taiwan | Fiber | 2011 | 7 |
| JX174433 | Taiwan | Fiber | 2011 | 7 |
| JX174434 | Taiwan | Fiber | 2011 | 7 |
| JX174435 | Taiwan | Fiber | 2011 | 7 |
| M23696 |  | Fiber |  | 7 |
| AY594255 |  | Fiber |  | 7 |
| AY921615 | USA | Fiber |  | 7 |
| GQ478341 | China | Fiber |  | 7 |
| HQ659699 | China | Fiber | 2007 | 7 |

| Accession numbers | Country | Gene | Year of the specimen collected | Serotype |
| --- | --- | --- | --- | --- |
| KC456083  (2902 – TWN) | Taiwan | Hexon | 2011 | 3 |
| KC456096  (1697 – TWN) | Taiwan | Hexon | 2011 | 3 |
| KC456089  (1012 – TWN) | Taiwan | Hexon | 2011 | 3 |
| KC456095  (1444 – TWN) | Taiwan | Hexon | 2011 | 3 |
| EF494645 | Taiwan | Hexon | 2003 | 3 |
| KC456092  (1225 – TWN) | Taiwan | Hexon | 2011 | 3 |
| KC456114  (1854 – TWN) | Taiwan | Hexon | 2011 | 3 |
| KC456103  (3759 – TWN) | Taiwan | Hexon | 2011 | 3 |
| EF494642 | Taiwan | Hexon | 1999 | 3 |
| KC456086  (552 – TWN) | Taiwan | Hexon | 2011 | 3 |
| EF494648 | Taiwan | Hexon | 2003 | 3 |
| KC456084  (198 – TWN) | Taiwan | Hexon | 2011 | 3 |
| KC456094  (1332 – TWN) | Taiwan | Hexon | 2011 | 3 |
| KC456093  (1301 – TWN) | Taiwan | Hexon | 2011 | 3 |
| KC456100  (1962 – TWN) | Taiwan | Hexon | 2011 | 3 |
| KC456085  (456 – TWN) | Taiwan | Hexon | 2011 | 3 |
| EF494646 | Taiwan | Hexon | 2003 | 3 |
| EF494647 | Taiwan | Hexon | 2003 | 3 |
| EF570053 | Taiwan | Hexon | 2003 | 3 |
| KC456091  (1133 – TWN) | Taiwan | Hexon | 2011 | 3 |
| KC456098  (1764 – TWN) | Taiwan | Hexon | 2011 | 3 |
| KC456102  (3440 – TWN) | Taiwan | Hexon | 2011 | 3 |
| KC456088  (889 – TWN) | Taiwan | Hexon | 2011 | 3 |
| EF494650 | Taiwan | Hexon | 2005 | 3 |
| KC456090  (1112 – TWN) | Taiwan | Hexon | 2011 | 3 |
| KC456097  (1735 – TWN) | Taiwan | Hexon | 2011 | 3 |
| KC456101  (3234 – TWN) | Taiwan | Hexon | 2011 | 3 |
| KC456087  (736 – TWN) | Taiwan | Hexon | 2011 | 3 |
| EF494644 | Taiwan | Hexon | 2000 | 3 |
| EF486500 | Taiwan | Hexon | 2000 | 3 |
| EF486502- | Taiwan | Hexon | 2002 | 3 |
| EF486503 | Taiwan | Hexon | 2002 | 3 |
| EF486505 | Taiwan | Hexon | 2005 | 3 |
| EF486501 | Taiwan | Hexon | 2000 | 3 |
| EF486504 | Taiwan | Hexon | 2003 | 3 |
| EF486506 | Taiwan | Hexon | 2002 | 3 |
| EF494643 | Taiwan | Hexon | 2004 | 3 |
| EF494640 | Taiwan | Hexon | 1992 | 3 |
| EF494641 | Taiwan | Hexon | 1996 | 3 |
| EF494649 | Taiwan | Hexon | 2003 | 3 |
| EF486496 | Taiwan | Hexon | 1996 | 3 |
| EF486497 | Taiwan | Hexon | 1996 | 3 |
| EF486498 | Taiwan | Hexon | 1997 | 3 |
| EF486499- | Taiwan | Hexon | 1999 | 3 |
| EF570054 | Taiwan | Hexon | 1983 | 3 |
| AY854178 | Korea | Hexon | 1999 | 3 |
| AY854173 | Korea | Hexon | 1991 | 3 |
| AY854175 | Korea | Hexon | 1992 | 3 |
| AY854176 | Korea | Hexon | 1998 | 3 |
| DQ099432 | China | Hexon | 2005 | 3 |
| AY878716 | China | Hexon |  | 3 |
| DQ105654 | China | Hexon | 2004 | 3 |
| AY854177 | Korea | Hexon | 1998 | 3 |
| AY854179 | Korea | Hexon | 1992 | 3 |
| AY854174 | Korea | Hexon | 1992 | 3 |
| AY854180 | Korea | Hexon | 1998 | 3 |
| AF542104 | Korea | Hexon | 1998 | 3 |
| AF542115 | Korea | Hexon | 1999 | 3 |
| AF542106 | Korea | Hexon | 1999 | 3 |
| AF542113 | Korea | Hexon | 1999 | 3 |
| AF542117 | Korea | Hexon | 1999 | 3 |
| AY599834 |  | Hexon |  | 3 |
| DQ086466 |  | Hexon |  | 3 |
| HQ659699 | China | Hexon | 2007 | 7 |
| AB330088 |  | Hexon |  | 7 |
| GQ478341 | China | Hexon | 2008 | 7 |
| AB243009 | Japan | Hexon | 2004 | 7 |
| AB243118- | Japan | Hexon | 2004 | 7 |
| AY594256 |  | Hexon |  | 7 |
| AF065066 | USA | Hexon | 1955 | 7 |
| JX423388 | USA | Hexon | 1997 | 7 |
| JN860676 | Argentina | Hexon | 1987 | 7 |
| AF065067 |  | Hexon |  | 7 |
| AF065068 | USA | Hexon | 1996 | 7 |
| AF515814 | China | Hexon |  | 7 |
| AY495969 | China | Hexon |  | 7 |
| AF053086 | Japan | Hexon | 1992 | 7 |
| AF053087 | Japan | Hexon | 1995 | 7 |
| AY769945 | Korea | Hexon | 1995 | 7 |
| AY769946 | Korea | Hexon | 1999 | 7 |
| JN860677 | USA | Hexon |  | 7 |
| KC456144  (1205 – TWN) | Taiwan | Hexon | 2011 | 7 |
| KC456145  (1777 – TWN) | Taiwan | Hexon | 2011 | 7 |
| KC456146  (1781 – TWN) | Taiwan | Hexon | 2011 | 7 |
| KC456147  (1796 – TWN) | Taiwan | Hexon | 2011 | 7 |
| KC456148  (1798 – TWN) | Taiwan | Hexon | 2011 | 7 |
| KC456149  (1825 – TWN) | Taiwan | Hexon | 2011 | 7 |
| KC456150  (1853 – TWN) | Taiwan | Hexon | 2011 | 7 |
| KC456151  (1873 – TWN) | Taiwan | Hexon | 2011 | 7 |
| KC456152  (1933 – TWN) | Taiwan | Hexon | 2011 | 7 |
| KC456153 (1965 – TWN) | Taiwan | Hexon | 2011 | 7 |
| KC456154  (2110 – TWN) | Taiwan | Hexon | 2011 | 7 |
| KC456155.  (2391 – TWN) | Taiwan | Hexon | 2011 | 7 |
| KC456156  (2394 – TWN) | Taiwan | Hexon | 2011 | 7 |
| KC456157  (2705 – TWN) | Taiwan | Hexon | 2011 | 7 |
| KC456143  (864 – TWN) | Taiwan | Hexon | 2011 | 7 |
| KC456158  (T60 – TWN) | Taiwan | Hexon | 2011 | 7 |
| KC456159  (T83 – TWN) | Taiwan | Hexon | 2011 | 7 |
| JX174426 | Taiwan | Hexon | 2011 | 7 |
| JX174427 | Taiwan | Hexon | 2011 | 7 |
| JX174428 | Taiwan | Hexon | 2011 | 7 |
| JX174429 | Taiwan | Hexon | 2011 | 7 |
| JX174430 | Taiwan | Hexon | 2011 | 7 |
| GU230898 | China | Hexon | 2009 | 7 |
| JF800905 | China | Hexon | 2009 | 7 |
| JQ360620 | China | Hexon | 2009 | 7 |
| JQ360621 | China | Hexon | 2011 | 7 |
| JQ360622 | China | Hexon | 2011 | 7 |
